# Validation of commercial Mas receptor antibodies for utilization in Western Blotting, immunofluorescence and immunohistochemistry studies

**DOI:** 10.1371/journal.pone.0183278

**Published:** 2017-08-16

**Authors:** Valeria Burghi, Natalia Cristina Fernández, Yamila Belén Gándola, Verónica Gabriela Piazza, Diego Tomás Quiroga, Érica Guilhen Mario, Janaína Felix Braga, Michael Bader, Robson Augusto Souza Santos, Fernando Pablo Dominici, Marina Cecilia Muñoz

**Affiliations:** 1 Universidad de Buenos Aires, Consejo Nacional de Investigaciones Científicas y Técnicas, Instituto de Química y Fisicoquímica Biológicas (IQUIFIB), Facultad de Farmacia y Bioquímica, Buenos Aires, Argentina; 2 Universidad de Buenos Aires, Consejo Nacional de Investigaciones Científicas y Técnicas, Instituto de Investigaciones Farmacológicas (ININFA), Facultad de Farmacia y Bioquímica, Buenos Aires, Argentina; 3 INCT-NanoBiofar, Department of Physiology and Biophysics, Biological Sciences Institute, Federal University of Minas Gerais, Belo Horizonte, Minas Gerais, Brazil; 4 Max-Delbrück Center for Molecular Medicine, Berlin, Germany; 5 Cardiology Institute of Rio Grande do Sul/University Foundation of Cardiology (IC/FUC), Porto Alegre, Rio Grande do Sul, Brazil; George Washington University School of Medicine and Health Sciences, UNITED STATES

## Abstract

Mas receptor (MasR) is a G protein-coupled receptor proposed as a candidate for mediating the angiotensin (Ang)-converting enzyme 2-Ang (1–7) protective axis of renin–angiotensin system. Because the role of this receptor is not definitively clarified, determination of MasR tissue distribution and expression levels constitutes a critical knowledge to fully understanding its function. Commercially available antibodies have been widely employed for MasR protein localization and quantification, but they have not been adequately validated. In this study, we carried on an exhaustive evaluation of four commercial MasR antibodies, following previously established criteria. Western Blotting (WB) and immunohistochemistry studies starting from hearts and kidneys from wild type (WT) mice revealed that antibodies raised against different MasR domains yielded different patterns of reactivity. Furthermore, staining patterns appeared identical in samples from MasR knockout (MasR-KO) mice. We verified by polymerase chain reaction analysis that the MasR-KO mice used were truly deficient in this receptor as *MAS* transcripts were undetectable in either heart or kidney from this animal model. In addition, we evaluated the ability of the antibodies to detect the human c-myc-tagged MasR overexpressed in human embryonic kidney cells. Three antibodies were capable of detecting the MasR either by WB or by immunofluorescence, reproducing the patterns obtained with an anti c-myc antibody. In conclusion, although three of the selected antibodies were able to detect MasR protein at high expression levels observed in a transfected cell line, they failed to detect this receptor in mice tissues at physiological expression levels. As a consequence, validated antibodies that can recognize and detect the MasR at physiological levels are still lacking.

## Introduction

Human Mas receptor (MasR) was described 30 years ago as a G protein–coupled receptor (GPCR) with transforming activity [1]. Currently, it is classified as a class A orphan GPCR [2]. More specifically, it was the founding member of the Mas-related GPCR subfamily (Mrgprs) consisting of mostly orphan receptors structurally homologous to MasR expressed in specific subpopulations of sensory neurons that detect painful stimuli [2]. In addition to the human gene, the *MAS* gene was isolated and characterized in rats and in mice. Highest levels of MasR transcripts were found in brain and testis, but lower levels were also detected in other organs such as heart and kidney [2–8]. The analysis of the human *MAS* cDNA sequence reveals an open reading frame that codes for a 325-amino-acid protein, whereas the mouse and rat homologues contain 324 amino acids [2, 6]. The predicted molecular weight (MW) of MasR is approximately 37 kDa (Uniprot: P04201, P30554 and P12526). The availability of MasR knockout (MasR-KO) mice facilitated the acquisition of information regarding the involvement of this receptor in behavioral, cardiovascular and renal processes [9, 10]. Noteworthy, multiple phenotypes are reported in literature for MasR-KO mice, showing both damaging and protective effects of the absence of MasR on organs and tissues [9].

MasR has been functionally and pharmacologically linked to angiotensin (Ang)-(1–7) [9, 11]. This heptapeptide is an endogenously produced renin–angiotensin system (RAS) hormone [12], mainly generated through the cleaving action of Ang-converting enzyme 2 (ACE2) on Ang II [13, 14]. The ACE2/Ang-(1–7) axis is involved in many physiologic and pathophysiological processes in several systems and organs, especially by opposing the detrimental effects of inappropriate overactivation of the classical RAS axis formed by ACE, Ang II, and Ang II type 1 (AT1) receptor [15]. Although MasR has been proposed as a candidate receptor for mediating the ACE2-Ang (1–7) protective axis of RAS, there are reports about the participation of the Ang II type 2 (AT2) receptor [16,17] and the AT1 receptor [18] in the actions of Ang-(1–7) as well as descriptions of other natural ligands for MasR, such as neuropeptide FF (NPFF) [19, 20], Ang III, Ang IV [21] and angioprotectin [22]. Thus, so far, it is not clear whether the interaction between Ang-(1–7) and MasR is mutually exclusive [9, 23].

As MasR has been suggested to participate in several physiological processes, the study of its function has generated increased interest due to potential therapeutic applications [9, 24]. Because its role has not been definitively clarified, sensitive and specific detection of the MasR protein is essential for studies aimed at elucidating its regulation in both physiological and pathophysiological conditions. Commercially available MasR antibodies have been widely used in investigations tending to determine MasR distribution, quantify its abundance and analyze its interaction with other molecules. Remarkably, the specificity of antibodies directed to several GPCRs has been questioned in many reports. This includes antibodies directed against various subtypes of α1- and β-adrenoceptors [25–27], muscarinic [27, 28], dopamine [29], galanin [30] as well as glucagon-like peptide-1 [31, 32] receptors. In many cases, multiple antibodies were used, and none of them were found to be selective. This indicated that, unfortunately, lack of specificity is frequent for GPCR antibodies [33]. More specifically, recent publications reported lack of specificity of commercially available AT1 [34, 35] and AT2 [36] receptor antibodies and the resulting difficulties in defining RAS receptors expression [37, 38].

During preliminary experiments using mice with targeted deletion of the *MAS* gene, we found contradicting results regarding the specificity of antibodies directed to MasR. As an adequate validation analysis was lacking, we carried out an exhaustive evaluation of a panel of commercial MasR antibodies. We followed a set of previously established validation criteria based in the principles of antibody action that establish a reasonable degree of assurance that the antibody being tested is actually targeting its correct antigen [33,34,36–42]. We focused on their utility for Western Blotting (WB), immunofluorescence (IF), and immunohistochemistry (IHC) studies. We employed mice genetically deficient in MasR that allowed a definitive evidence of antibody specificity, and also evaluated the ability of these antibodies to detect different expression levels of the receptor through the use of a heterologous cell line overexpressing the human MasR.

## Materials and methods

### Antibodies

**Fig 1:**
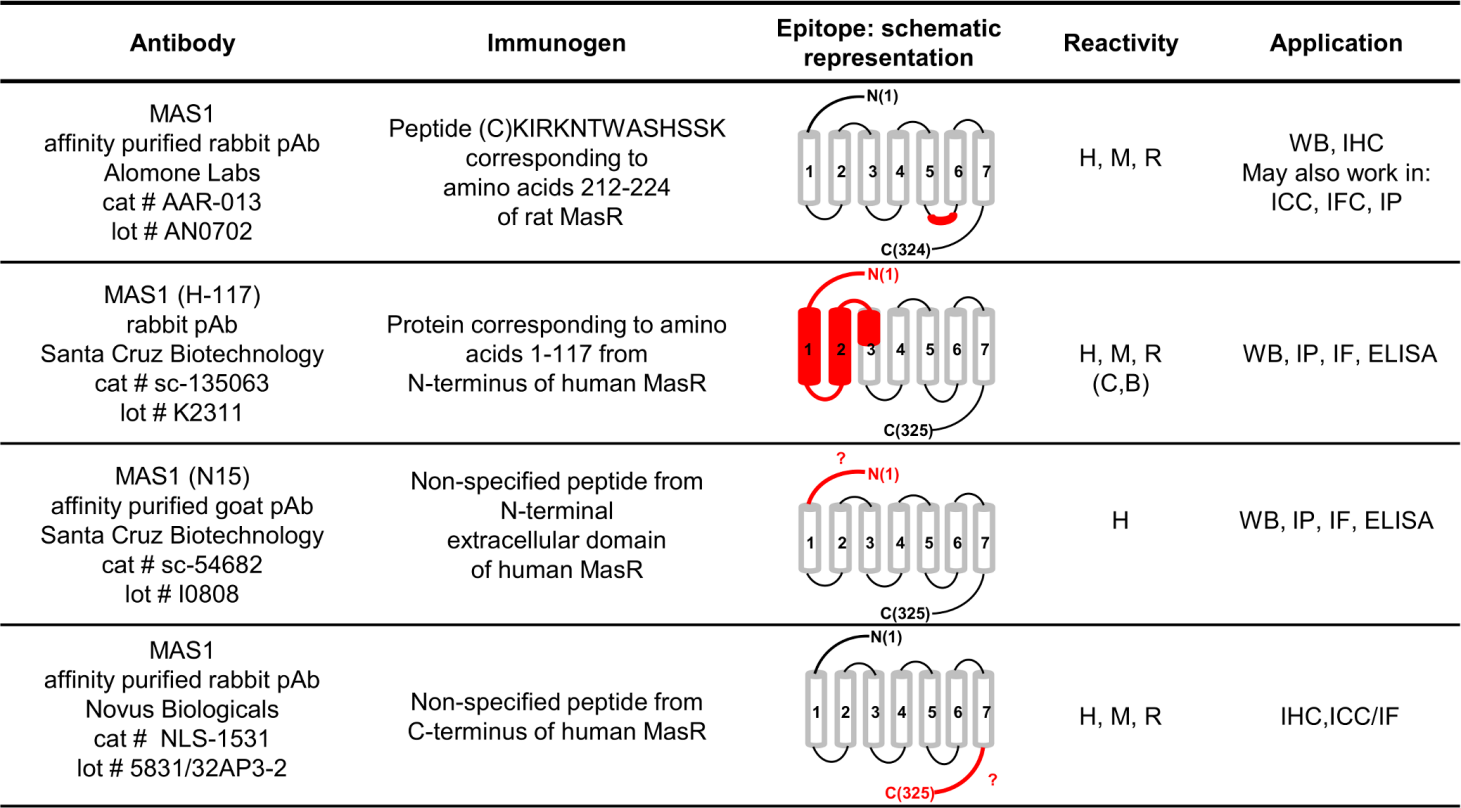
Characteristics of MasR antibodies used in the study. pAb polyclonal antibody, H human, M mouse, R rat, C canine, B bovine, WB western blot, IHC immunohistochemistry, ICC immunocytochemistry, IF immunofluorescence, IP immunoprecipitation, IFC indirect flow cytometry, ELISA enzyme-linked immunosorbent assay.

Four commercial antibodies raised against different domains of the MasR were selected for their validation. Two antibodies (sc-135063 and sc-54682) were purchased from Santa Cruz Biotechnology (Santa Cruz, CA, USA); AAR-013 was from Alomone Labs (Jerusalem, Israel) and NLS1-1531 was from Novus Biologicals (Littleton, CO, USA). During the last phase of this study Santa Cruz Biotechnology discontinued a large number of its polyclonal products including sc-135063 and sc-54682 antibodies. The information of immunogen used, reactivity and applications as provided by the manufacturers is presented in Fig 1.

### Animals

To obtain Mas receptor knockout (MasR-KO) animals on a pure genetic background, MasR^+/-^ mice (mixed genetic background, 129 x C57BL/6) [10] were bred to the FVB/N mouse line (Charles River, Sulzfeld, Germany) for seven generations at the Max Delbrück Center for Molecular Medicine. The selection for the MasR-KO allele was done by polymerase chain reaction (PCR) with primers MAS12: 5´-GCC GTT GCC CTC CTG GCG CCT GGG-3´ and NeoPVU: 5´-GGC AGC GCG GCT ATC GTG G-3´. Primers MAS10: 5´-GTA CAG CTT CGA AGA ATG GGA GGC CC-3´ and MAS14: 5´-CCT AAC TGA GCC ACC CTC ACC-3´ were used for the detection of wild-type alleles. Thereafter, F7 heterozygous males were bred with F7 heterozygous females to generate the line FVB/N MasR-KO. Mice were maintained at the transgenic animal facilities of the Laboratory of Hypertension (Federal University of Minas Gerais, Belo Horizonte, Brazil) and were treated according to the international guidelines for animal care. Nine-week-old male FVB/N wild type (WT) and FVB/N MasR-KO mice were used for the experiments. The experimental protocol was approved by the ethics committee in animal experimentation of the Federal University of Minas Gerais (protocol no. 006/05). The animals were maintained under controlled light and temperature conditions and had free access to water and standard diet.

### Molecular construct

We used a MasR expression construct that consisted of the cDNA sequence corresponding to the human MasR (accession number: NM_002377.2) fused to the c-myc tag peptide at N-terminal. The construct was synthesized and subcloned into the pcDNA 3.1 (+) mammalian expression vector between BamHI and EcoRI restriction sites by Life Technologies (Grand Island, NY) (pcDNA 3.1/c-myc-MasR). An empty vector [pcDNA 3.1 (+)] was used both as control and to deliver an equal amount of plasmid per transfection in Western Blotting (WB) and in immunofluorescence (IF) experiments.

### Cell culture and transient transfections

Human embryonic kidney (HEK293T) cells were obtained from the American Type Culture Collection (ATCC, Manassas, VA). They were cultured in Dulbecco's modified Eagle's medium (DMEM) supplemented with 10% fetal bovine serum (FBS), 2 mM glutamine and 5 μg/ml gentamicin and maintained at 37°C in a humidified atmosphere containing 5% CO_2_. For transient transfections, the cDNA constructs were transfected using Lipofectamine 2000 (Life Technologies). The transfection protocol was optimized as recommended by the suppliers. For IF experiments, approximately 2 x 10^4^ cells were seeded on 0.01% (w/v) poly-L-lysine pre-treated 15 mm coverslips and grown for 24 h. Transient cotransfections were performed with 0.25 μg pcDNA 3.1/c-myc-MasR and 0.25 μg empty plasmid pcDNA 3.1. Cells transfected with 0.5 μg of empty plasmid pcDNA 3.1 were used as controls. For WB experiments, approximately 5 x 10^5^ cells were seeded in 12-well culture plates and the following day were transfected with 0, 0.2, or 0.5 μg of pcDNA 3.1/c-myc-MasR. Empty plasmid pcDNA 3.1 was utilized to deliver an equal amount of plasmid per transfection.

### Protein extraction from rodent tissues and cultured cells

WT and MasR-KO mice were anesthetized by the intraperitoneal administration of a mixture of ketamine and xylazine (60 and 10 mg/kg, respectively), and submitted to the surgical procedure as soon as anesthesia was assured by the loss of pedal and corneal reflexes. The abdominal cavity was opened and the heart and kidney were removed. Tissue were homogenized at the ratio 0.1 g/1 ml in a buffer containing 1% Triton, 100 mM Hepes, 100 mM sodium pyrophosphate, 100 mM sodium fluoride, 10 mM EDTA, 10 mM sodium vanadate, 2 mM phenylmethylsulfonyl fluoride (PMSF) and 0.035 trypsin inhibitory units/ml aprotinin (pH 7.4) at 4°C. Resulting homogenates were centrifuged at 15700 g for 40 min at 4°C to remove insoluble material. Protein concentration in the supernatant was determined by the BCA assay (BCA Protein Assay Reagent, Thermo Scientific Pierce). Equal amount of tissue proteins were denatured by boiling 5 min in Laemmli buffer and stored at -20°C until electrophoresis. HEK293T cells were lysed 48 h after transfection at room temperature in Laemmli buffer and sonicated to shear DNA. Heat treatment of samples was avoided to prevent aggregation of overexpressed MasR.

### Western blotting

The protein extracts were subjected to SDS–PAGE using a Mini Protean apparatus (Bio-Rad Laboratories, PA, USA) and transferred to polyvinylidenedifluoride (PVDF) membranes. For heart and kidney tissue, 40 μg of protein homogenates were loaded onto the polyacrylamide gel whereas for HEK293T cells 25 μg were used.

To reduce non-specific antibody binding, membranes were incubated for 2 h at room temperature in bovine serum albumin (BSA)-based blocking buffer. The membranes were then exposed to primary antibodies overnight at 4°C. Only those antibodies recommended for WB (Fig 1) were tested. Based on the described reactivity we used AAR-013 (1:5000) and sc-135063 (1:1000), for detection of the MasR present in mouse tissues, while AAR-013, sc-135063 and sc-54682 1:1000 were used for detection of the human receptor expressed in HEK293T cells. Anti c-myc tag antibody (Cell Signaling Technology Inc.; cat. #2272) diluted 1:1000 was used to detect the c-myc tagged MasR. Anti-β-tubulin (Abcam Inc.; cat. #Ab6046) diluted 1:5000 was used as a protein loading control. After incubation for 1 h at room temperature using a 1:20000 dilution of the appropriate horseradish peroxidase-conjugated secondary antibody, sc-2004 (anti-rabbit) and sc-2020 (anti-mouse) (Santa Cruz Biotechnology, CA, USA), reaction products were revealed by enhanced chemiluminescence (ECL Plus, Pierce, Rockford, IL, USA).

### Immunohistochemical staining

Heart and kidneys from MasR-KO and WT mice were prepared by immersion fixation in 10% buffered formalin, processed for paraffin embedding, and sectioned at a thickness of 4 μm. Deparaffinized tissue sections were then rehydrated and subjected to antigenic recuperation with sodium citrate buffer (pH 6.0) at 98°C for 30 min. Endogenous peroxidase activity was blocked by incubating slides on PBS containing 3% hydrogen peroxide for 30 min. Non-specific protein binding was blocked by incubation with PBS containing 1% BSA for 1 h followed with normal horse serum for 2 h. Heart and kidney sections were incubated with anti-MasR antibodies recommended for immunohistochemistry [AAR-013 (1:300) and NLS-1531 (1:100)] overnight at 4°C. Preadsortion control of AAR-013 antibody with the blocking peptide provided by the manufacturers was performed overnight at 4°C using 10 μg peptide per 1 μg antibody. Negative controls were performed in parallel by replacing the primary antibody by PBS containing 1% BSA. Subsequently, incubations with biotin-labeled secondary antibodies followed by incubation with streptavidin-horse radish peroxidase complex were each performed for 30 min at room temperature (R.T.U. vectastain kit, Vector Laboratories, CA, USA). The antigen-antibody binding was visualized with diaminobenzidine (DAB) (Peroxidase substrate kit, DAB sk-4100, Vector Laboratories), and sections were counterstained with hematoxylin. Immunostained sections were observed under light microscopy using a Leica DM2000 microscope; representative color photomicrographs were obtained at 40x magnification using a Leica DFC400 digital camera and Leica Application Suite software (Leica Microsystems).

### Immunofluorescence

HEK293T cells were washed with PBS 24 h after transfection, fixed in 2% formaldehyde in PBS for 10 min and permeabilized with 0.2% Triton X-100 in PBS for 15 min. The non-specific binding sites were blocked for 30 min with blocking solution (PBS containing 1% BSA, 0.2% Triton-X-100). Then cells were incubated for 1 h at room temperature with the anti-c-myc antibody (1:200) or with four different anti-MasR antibodies that were recommended for immunocytochemistry [NLS-1531, sc-135063 and sc-54682, (diluted 1:200) and AAR-013 (diluted 1:100)]. All dilutions were in the range recommended by the manufacturers. Following washing with PBS, cells were incubated with secondary cyanine (Cy3)-conjugated antibody 1:200 for 1 h at room temperature. Donkey Cy3-conjugated IgG (Jackson ImmunoResearch Laboratories Inc., West Grove, PA) cat # 711-165-152 and # 705-165-003 secondary antibodies were used to detect rabbit or goat primary antibodies, respectively. After washing with PBS, the cells were incubated with Hoechst 33258 nucleic acid stain (5 *μ*g/mL) for 10 min. Finally, the coverslips were washed with PBS and mounted on glass slides with mounting medium. Images were acquired using a spectral laser scanning confocal Zeiss AxioObserver Z1 LSM710 fluorescent microscope (40X apochromatic, 1.4 NA objective) using dual excitation (543 nm for Cy3 and 405 nm for Hoechst). Controls prepared by omission of primary antibodies did not show any Cy3 fluorescence under the above conditions.

### RNA isolation and RT-PCR

Total RNA was extracted from heart and kidneys from MasR-KO and WT mice using TRIzol Reagent (Ambion ®) and then 2 μg of total RNA previously treated with DNase I Amplification Grade (Invitrogen^TM^, CA, USA) were subjected to reverse transcription using iScript^TM^ cDNA Synthesis Kit (Bio-Rad Laboratories, Inc.) according to manufacturer´s instructions. PCR reactions were carried out in a 50 μl reaction volume containing the following reagents: 1 μl of cDNA preparation; 1X Green GoTaq^®^ Reaction Buffer (Promega); 200 μM of dNTP Mix (Invitrogen); 1,25 U of GoTaq^®^ DNA polymerase (Promega), and 0.4 μM of forward and reverse primers. PCR was performed on a MasterCycler (Eppendorf, Hamburg, Germany), using the following conditions for denaturation, annealing, and extension (35 cycles): 94°C for 30 s, 54°C for 30 s, and 72°C for 40 s, followed by 72°C for 5 min. The specific primers sequences were as follows: for MasR 5´-GGA GAA ATC CCT TCA CGG TC-3´ and 5´-GGA CAC TAA CAT GAG CGG AG-3´ (Amplicon size: 434 bp); for 18S rRNA 5´-ACG GAC AGG ATT GAC AGA TT-3´ and 5´-GCC AGA GTC TCG TTC GTT AT-3´ (Amplicon size: 118 bp). PCR products were separated by electrophoresis on a 2% agarose gel and visualized after staining with ethidium bromide (0.5 μg/mL).

## Results

### Detection of MasR mRNA in heart and kidney from wild type and MasR-KO mice

**Fig 2:**
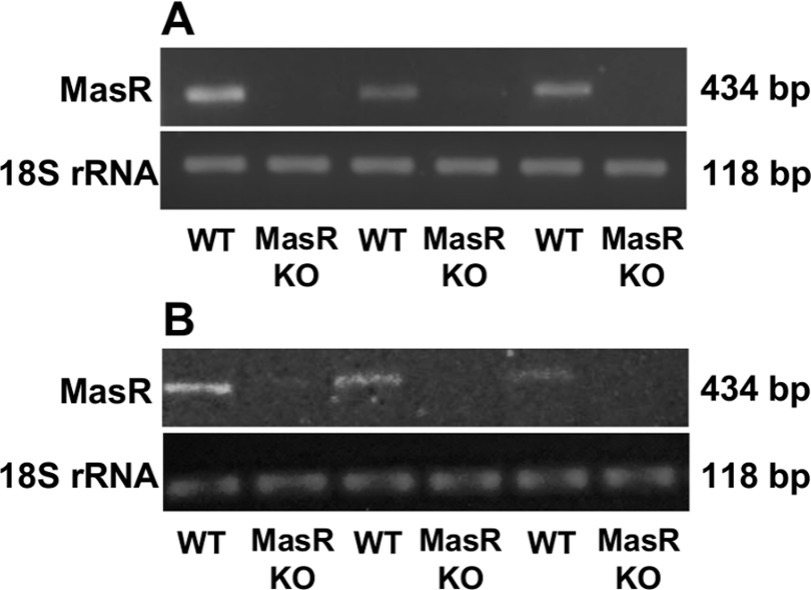
Detection of MasR mRNA in heart and kidney from wild type and MasR-KO mice. Representative agarose gel (n = 3–5) showing reverse transcription polymerase chain reaction (RT-PCR) product obtained from hearts (A) and kidneys (B) of wild type (WT) and MasR-KO mice. MasR mRNA was undetectable in heart and kidney of MasR-KO mice. 18S ribosomal RNA (18S rRNA) was used as housekeeping gene.

To verify the absence of MasR in the knockout mice used in these studies, we measured the amount of MasR mRNA in hearts and kidneys from MasR-KO mice. Reverse transcription polymerase chain reaction (RT-PCR) analysis showed that MasR mRNA was present in hearts and kidneys from wild type (WT) mice (Fig 2). In contrast, MasR transcript was undetectable in either heart (Fig 2A) or kidney (Fig 2B) from MasR-KO mice.

### Evaluation of MasR antibodies by Western blotting of heart and kidney from wild type and MasR-KO mice

**Fig 3:**
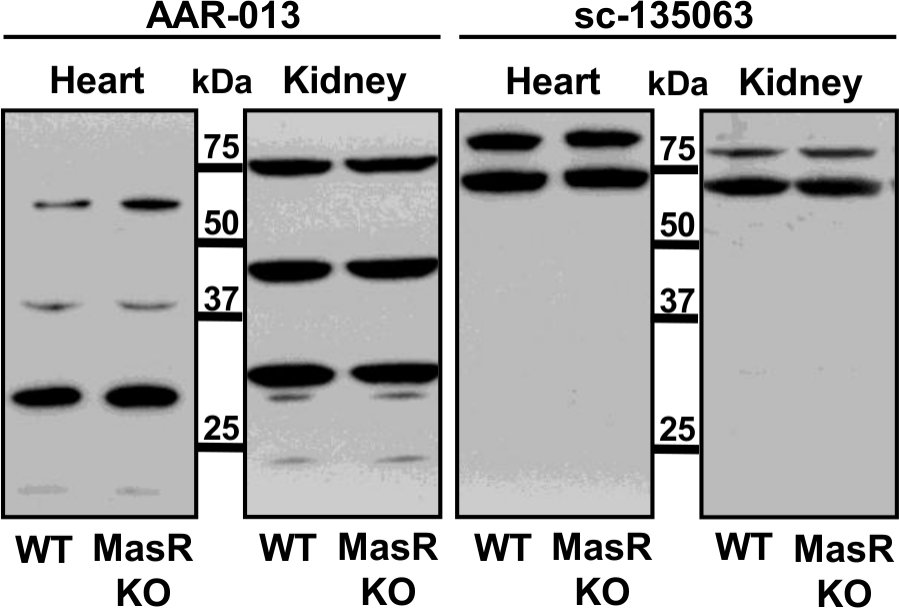
Western blot analysis of heart and kidney homogenates from wild type (WT) and MasR-KO mice. The predicted molecular weight of MasR is about 37 kDa. As observed, the assayed antibodies generated different immunoreactivity patterns. In both cases there were no differences in the band patterns obtained with tissues from WT and MAS-KO mice. The membranes are representative of n = 4.

We performed a WB analysis using hearts and kidneys protein extracts from WT and MasR- KO mice. Band patterns produced by two antibodies intended for detection of MasR of mouse origin in WB studies were compared. As shown in Fig 3, antibody AAR-013 generated three bands when exposed to heart homogenates of WT mice. The most prominent one of a MW ranging from 25 to 37 kDa, another of much lower intensity just above 37 kDa and the third one between 50 and 75 kDa (Fig 3). When using kidneys homogenates of the same animals, the AAR-013 yielded three bands of similar intensity, one of a MW ranging from 25 to 37 kDa, another close to 50 kDa and the third one in the range of 75 kDa (Fig 3). Using the same samples, antibody sc-135063 generated two bands of high molecular mass, at about 75 kDa (Fig 3). Thus, it was found that these two antibodies raised against different MasR domains revealed different patterns of reactivity. Importantly, none of them detected a single band, and only antibody AAR-013 generated a band of approximately the expected 37 kDa size of the MasR (Fig 3). Strikingly, in both cases there were no apparent differences in the pattern of reactivity when hearts and kidneys homogenates from WT and MasR-KO mice were compared (Fig 3).

### Evaluation of MasR antibodies by immunohistochemistry of heart and kidney from wild type and MasR-KO mice

**Fig 4:**
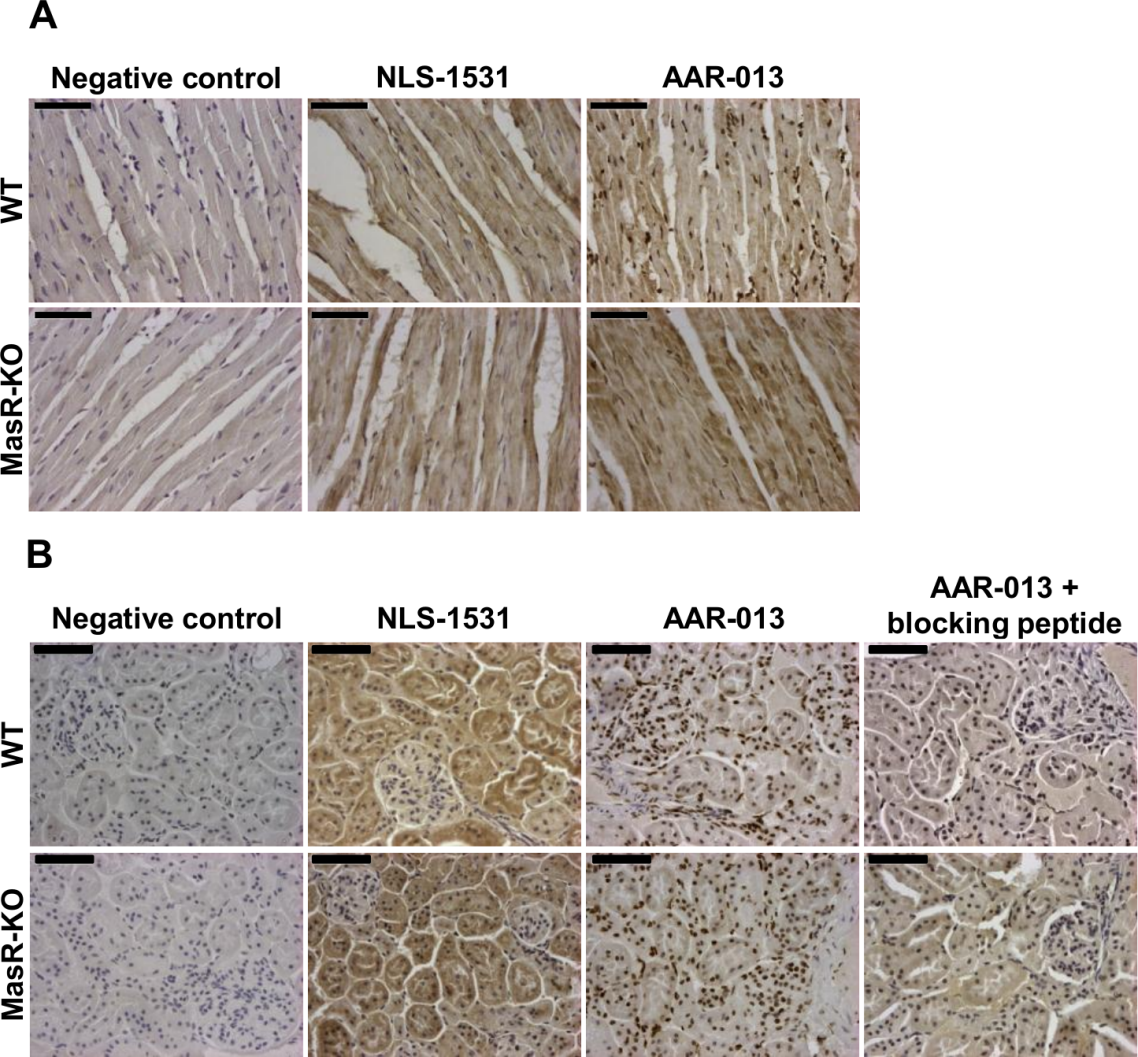
Immunohistochemistry in heart and kidney from wild-type (WT) and MasR-KO mice. Negative controls performed by omitting the primary antibody demonstrated minimal background immunostaining in heart (A) and kidney (B) sections. (A) In heart sections, NLS-1531 antibody stained predominantly the cytoplasm of cardiomyocytes with similar intensity in WT and MasR-KO hearts. The AAR-013 antibody stained the cardiomyocytes nucleus and weaker staining was observed in their cytoplasm. The same pattern was found in heart sections of MasR-KO mice. (B) In kidney sections, for NLS-1531 antibody, staining was mostly restricted to cytoplasm of numerous tubules cells with similar intensity in WT and MasR-KO kidneys. Antibody AAR-013 stained most intensely tubules cells nucleus and weaker staining was observed in their cytoplasm. Glomeruli were also stained positively. The same pattern was found in kidney sections of MasR-KO. Preincubation of the AAR-013 antibody with the blocking peptide provided by the vendors eliminated the immunohistochemical nuclear staining both in WT and MasR-KO mice kidney sections. Images are representative of n = 3. Bar, 50 μm.

Two antibodies recommended for detection of MasR of mouse origin by IHC were tested. As shown in Fig 4, tissue sections from hearts and kidneys demonstrated minimal background staining in the absence of primary antibody, compared with sections incubated with the MasR antibodies. In heart sections of WT mice (Fig 4A), NLS-1531 antibody stained predominantly the cytoplasm of cardiomyocytes, attaining similar intensity in WT and MasR-KO hearts (Fig 4A). The AAR-013 antibody generated an intense staining in the nuclei of cardiomyocytes, a weaker staining was detected in their cytoplasm. Of note, a similar pattern of staining was also found in heart sections of MasR-KO mice (Fig 4A). In kidney sections of WT mice (Fig 4B), NLS-1531 antibody staining of WT kidney sections was mostly restricted to cytoplasm of tubules cells. Strikingly, incubation of NLS-1531 antibody with kidney sections of MasR-KO mice lead to similar results (Fig 4B). When analyzing the reactivity of the AAR-013 antibody, we found staining in tubules and glomeruli, with more pronounced nuclear staining and lesser staining in the cytoplasm (Fig 4B). Although we showed that MasR was absent in MasR-KO, a similar staining was detected when kidney sections of MasR-KO mice were incubated with AAR-013 antibody (Fig 4B). Preincubation of the antibody with the blocking peptide provided by the manufacturers abrogated the nuclear staining in kidney sections from both WT and MasR-KO mice.

### Evaluation of MasR antibodies by immunofluorescence of HEK293T cells overexpressing MasR

**Fig 5:**
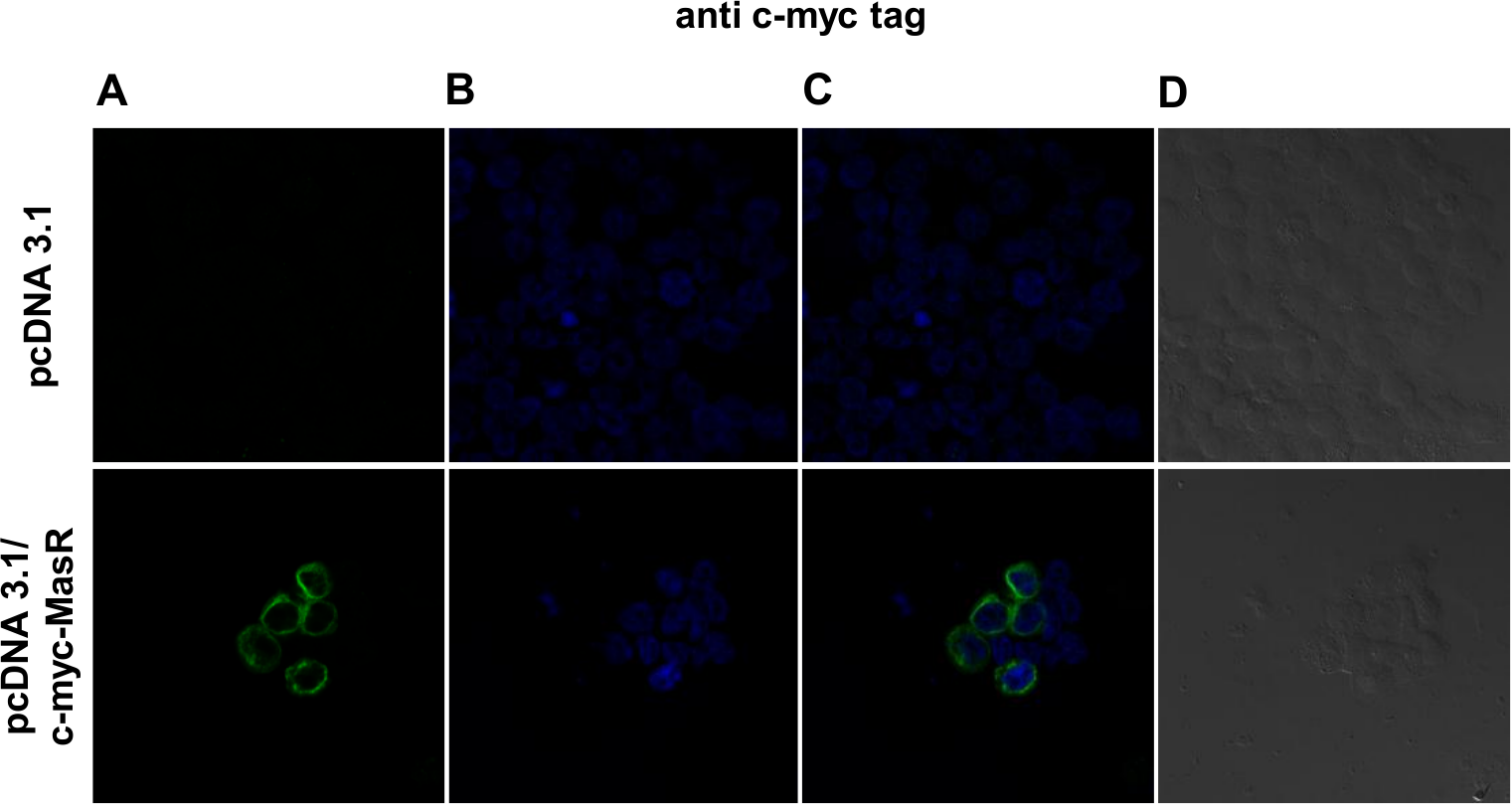
Immunofluorescence studies in HEK293T cells overexpressing c-myc tagged MasR using anti c-myc tag antibody. Images of fluorescence signal corresponding to secondary cyanine (Cy3)-conjugated antibody (A), nuclear Hoechst 33258 staining (B), merged images (C) and bright field (D) are shown. The intense immunostaining obtained with the anti c-myc tag antibody indicated strong expression of MasR in HEK293T cells after transfection with the pcDNA 3.1/c-myc-MasR construct. A significant amount of the total MasR pool was present at the plasma membrane. No c-myc staining was observed in cells transfected with the empty vector pcDNA 3.1. Images are representative of 3 independent experiments.

To explore the possibility that the above commented results were a consequence of low sensitivity, we overexpressed the MasR in human embryonic kidney (HEK293T) cells by transfecting them with DNA encoding the human MasR. Total expression and cellular distribution of MasR protein was evaluated by fluorescence confocal microscopy. We expanded the possibilities of its immunological detection by using a plasmid encoding the MasR fused to the N-terminal c-myc tag. In addition, we used cells transfected with the empty vector as a control. As shown in Fig 5 an intense c-myc immunostaining was obtained 24 h after transfection, indicating that the MasR was strongly expressed. A significant amount of the total MasR pool was present at the plasma membrane. However, a non-plasma membrane-associated MasR pool was also detectable, especially in cells showing higher levels of receptor expression. Noticeably, no intranuclear localization of MasR was detected. No c-myc staining was found in cells that had been transfected with the empty vector.

**Fig 6:**
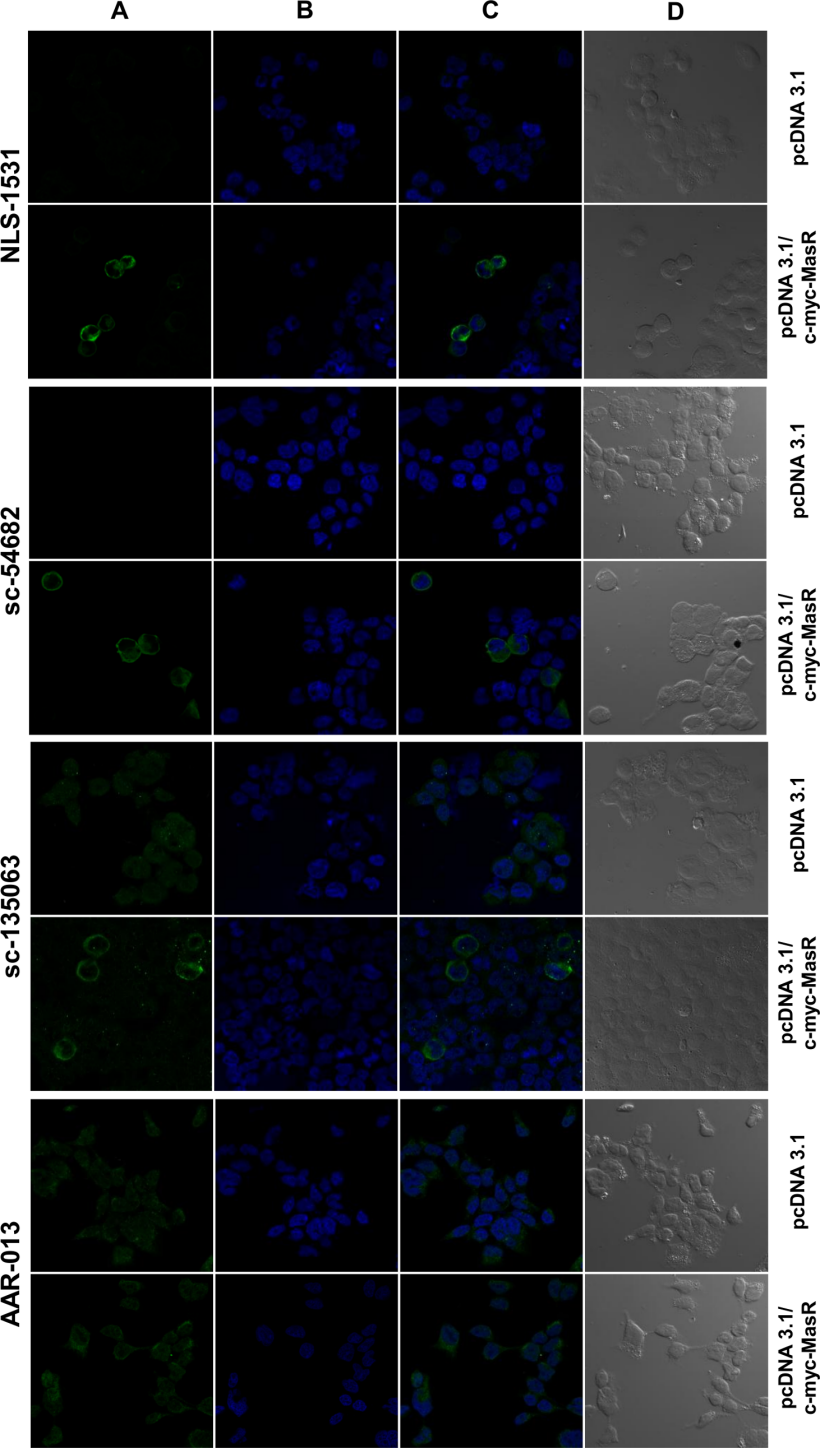
Immunofluorescence studies in HEK293T cells overexpressing c-myc tagged MasR using MasR antibodies. Images of fluorescence signal corresponding to secondary cyanine (Cy3)-conjugated antibody (A), nuclear Hoechst 33258 staining (B), merged images (C) and bright field (D) are shown. In cells transfected with the pcDNA 3.1/c-myc-MasR construct, antibodies NLS-1531 and sc-54682 generated a similar staining pattern to that generated with the anti c-myc antibody. No staining was observed with any of these antibodies in cells transfected with the empty vector pcDNA 3.1. Antibody sc-135063 was capable of staining the plasma membrane of cells overexpressing the MasR but also generated widespread signals in cells transfected with the empty vector. For antibody AAR-013 there was no staining associated with the cell membrane. Intense immunocytochemical staining of identical distribution and intensity was revealed in cells transfected with the empty vector and those transfected with the c-myc-MasR construct. Images are representative of 3 independent experiments.

Next we evaluated the reactivity of four antibodies, suggested for utilization in IF analysis, towards the human MasR. As shown in Fig 6, antibodies NLS-1531 and sc-54682 generated a similar staining pattern to that obtained with incubation with the anti c-myc antibody. Both of them stained predominantly the plasma membrane and also the perinuclear area. No staining was observed for cells that had been transfected with the empty vector. Antibody sc-135063 also stained the plasma membrane of cells overexpressing MasR. (Fig 6). However, it also generated widespread signals of lower intensity in overexpressing cells and in cells transfected with the empty vector. Finally, antibody AAR-013 did not generate any staining associated with the cell membrane while it revealed intense immunocytochemical staining of identical distribution and intensity in cells transfected with the empty vector and those transfected with the pcDNA 3.1/c-myc-MasR construct (Fig 6).

### Evaluation of MasR antibodies by Western blotting of HEK293T cells overexpressing MasR

**Fig 7:**
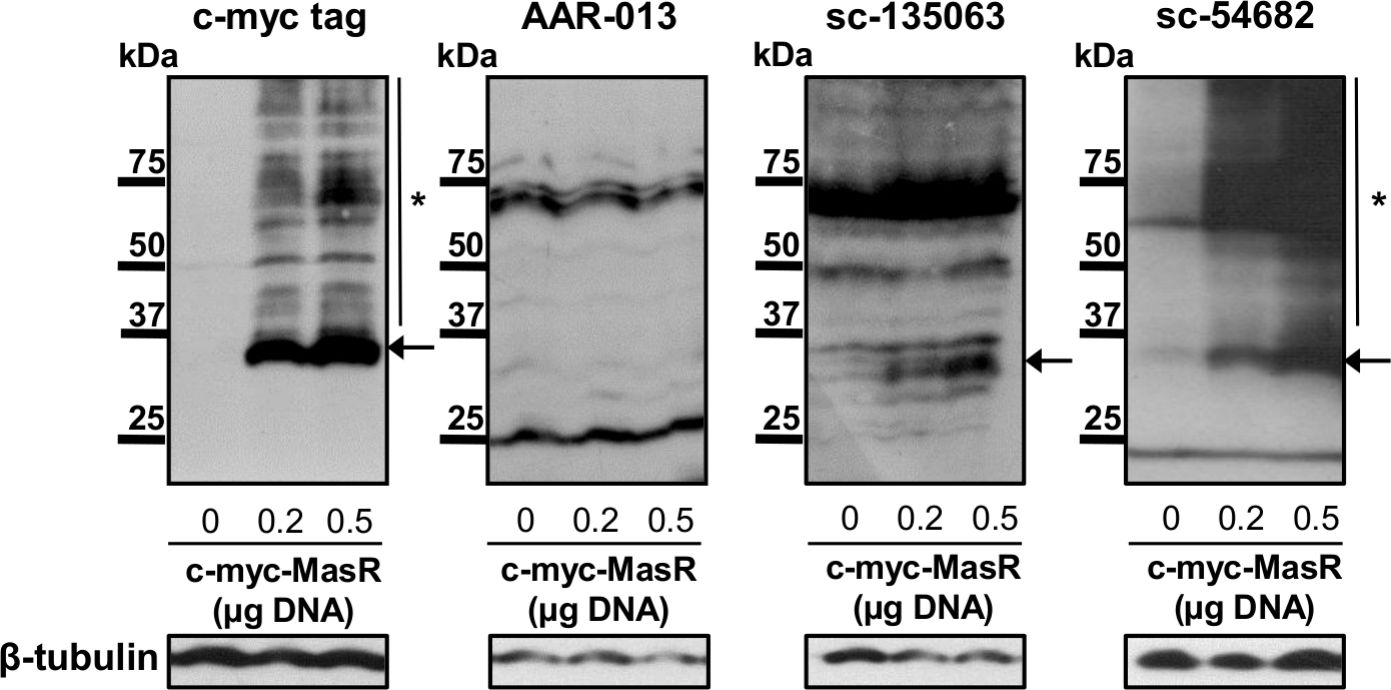
Western blotting of HEK293T cells overexpressing c-myc tagged MasR using anti c-myc tag and three MasR antibodies. Cells were transfected with varying amounts of a plasmid encoding the c-myc-tagged MasR (0.2 and 0.5 μg of pcDNA 3.1/c-myc-MasR).The anti-c-myc antibody detected a band of 37 kDa whose intensity increased as higher amount of DNA was transfected (arrow). Additionally, a ladder of higher molecular weight (MW) immunoreactive bands could be observed (asterisk). sc-54682 antibody reproduced the band pattern obtained with the c-myc antibody (arrow and asterisk). sc-135063 antibody revealed multiple bands close to 37 kDa with one of them of increasing intensity as higher amount of DNA was transfected (arrow) but did not produce a ladder of higher MW immunoreactive bands. High unspecific staining was attained with sc-135063 antibody. AAR-013 antibody revealed a pattern of immunoreactivity not comparable to those obtained with the other antibodies. Detection of β-tubulin was used as a protein loading control. The membranes are representative of 3 independent experiments.

We also evaluated the ability of the commercial antibodies to detect MasR protein increments at different levels of overexpression. To that end, HEK293T cells were transfected with increasing amounts of pcDNA 3.1/c-myc-MasR. Cell proteins were solubilized and subjected to WB. Results are presented in Fig 7. The anti-c-myc antibody revealed a prominent band of a slightly lower MW than the predicted 37 kDa for the MasR in protein extracts from cells transfected with 0.2 μg pcDNA 3.1/c-myc-MasR. In protein extracts from cells transfected with 0.5 μg pcDNA 3.1/c-myc-MasR, a detectable increase in the intensity of this band was observed (Fig 7). In addition, a ladder of higher MW immunoreactive bands was also detected (Fig 7).

Next we evaluated if the band pattern obtained with the anti-c-myc tag antibody could be reproduced by three commercial antibodies against human MasR suggested for WB. Antibody AAR-013 revealed a very different pattern of immunoreactivity, a faint band of approximately 37 kDa was detected. Instead, this antibody yielded two prominent bands of both higher (75 kDa) and lower (25 kDa) molecular size (Fig 7). Bands intensity was similar although increasing amounts of pcDNA 3.1/c-myc-MasR were transfected. Antibody sc-135063 revealed multiple bands of MW close to 37 kDa in protein extracts from cells transfected with 0.2 μg pcDNA 3.1/c-myc-MasR. Notably, an increase in the intensity of one of them was observed when cells were transfected with 0.5 μg pcDNA 3.1/c-myc-MasR (Fig 7). Unlike results obtained with the anti c-myc tag antibody, sc-135063 antibody did not produce a ladder of immunoreactive bands of higher MW. Instead, it generated bands of higher MW (50 and 75 kDa respectively) that had similar intensity regardless of the amount of pcDNA 3.1/c-myc-MasR plasmid used for transfection (Fig 7). Finally, sc-54682 antibody generated a similar band pattern to that obtained with the anti-c-myc antibody. As shown in Fig 7, sc-54682 antibody revealed a band of around 37 kDa in protein extracts from cells transfected with 0.2 μg pcDNA 3.1/c-myc-MasR. The intensity of this band increased when cells were transfected with 0.5 μg pcDNA 3.1/c-myc-MasR (Fig 7). Similarly to the pattern obtained with the anti c-myc antibody, sc-54682 generated a ladder of immunoreactive bands of higher MW. We observed an additional band above 50 kDa, whose intensity remained unaltered despite transfecting increasing amounts of pcDNA 3.1/c-myc-MasR.

## Discussion

During the last two decades, antibodies towards MasR have been widely used in scientific reports aimed to understand its molecular regulation in a variety of physiological and pathophysiological conditions. At the same time, uncertainties about the usefulness of several GCPRs antibodies, such as those against AT1 and AT2 receptors have been raised [25–36]. As an adequate validation analysis was lacking, we carried on an evaluation of four commercial MasR polyclonal antibodies following previously established criteria. During the last phase of this study, Santa Cruz Biotechnology discontinued a large number of its polyclonal products including two of the four assayed antibodies. However, these Santa Cruz polyclonal antibodies may still be available in several research laboratories. More importantly, a validation analysis is crucial to interpret previously published work using them.

A major criterion in locating a valid antibody is that the precise antigen sequence against which the antibody was raised should be provided. Full information for each antibody should be known and included into the methods section of scientific publication to assure that the experiments are repeatable [39–42]. Two of the MasR antibodies studied, AAR-013 and sc-135063, raised against different domains of the receptor, met this criterion (Fig 1). The other two antibodies (sc-54682 and NLS-1531), were provided without the exact sequence of the immunizing peptide but with a statement that they were raised against part of the N- terminal and C-terminal domain, respectively.

Immunoblotting provides a sensitive and discriminating method for antibody validation because it clearly distinguishes crossreacting proteins of different molecular sizes. In tissues expressing the MasR, antibodies should detect a single band at the predicted MW (∼37 kDa). For our study we used two rodent tissues (heart and kidney) which are known to express the MasR. None of the two antibodies intended for detection of MasR of mouse origin in WB studies, sc-135063 and AAR-013 antibodies, detected a single band, and only AAR-013 generated a band of approximately the expected 37 kDa size of the receptor. The detection of multiple bands or bands of a MW different from the predicted, could be indicative of recognition of MasR in different states of posttranslational modifications, in oligomeric forms or breakdown products. In any case, the use of such an antibody requires verification with antibodies against a different part of the molecule to demonstrate that the staining is genuine [39]. In each tissue, the two mentioned antibodies raised against different MasR domains revealed different patterns of reactivity. The high variability on the ability of the different antibodies to recognize these bands raised the concern that each antibody binds to distinct unknown proteins of diverse molecular sizes other than the MasR.

The reactivity of a given antibody with its target protein depends, among other factors, on the abundance of that protein in the preparation [42]. Accordingly, we performed the most stringent control for antibody specificity that requires comparison of antibody reactivity in WT tissues with low physiological receptor expression densities to reactivity in KO animals in which endogenous MasR protein expression has been genetically deleted. We verified by RT-PCR analysis that the MasR-KO mice used in the current study were truly deficient in this receptor, as *MAS* transcripts were undetectable in both the heart and the kidney of this animal model. Strikingly, the two analyzed antibodies showed the same pattern of bands when hearts and kidneys homogenates from WT and MasR-KO mice were compared. These results indicate that the immunoreactivity observed is not related to the presence or absence of the MasR and that each of these antibodies react with multiple off-targets proteins. In MasR-KO mice, the *MAS* gene was disrupted by insertion of a neomycin resistance cassette resulting in deletion of the region coding for the amino-terminal 253 amino acids of the receptor including six transmembrane domains [10]. Therefore, it is possible that some antibodies may interact with non-functional fragments of the protein potentially expressed in MasR-KO mice. However, we believe this would not be the case, considering that AAR-013 and sc-135063 antibodies were raised against amino acids 212–224 and 1–117, respectively. These sequences correspond to domains of the MasR that were untranslated in the MasR-KO mice used. The identity of the proteins recognized by these antibodies remains unknown. Our study design does not allow us to rule out that the bands yielded by the antibodies correspond to related proteins, structurally homologous to MasR that could potentially be upregulated in MasR-KO mice. In summary, results obtained from these experiments suggested that the analyzed MasR antibodies were unable to detect the endogenous MasR in heart and kidney from mice using conditions traditionally employed for conventional WB analysis. This conclusion is directly applicable only for the mentioned mice tissues. Staining in tissues from other species may still represent other antigens (not present in mice) and hence require additional controls.

While this type of antibody validation was a useful first step, it only provided data for WB studies. There is no absolute correlation between the specificity of the labeling in WB and IHC, so it was necessary to demonstrate the usefulness of MasR antibodies for this latter application. Accordingly, we performed IHC studies using antibodies AAR-013 and NLS-1531 that were recommended by the vendors for this application. In heart and kidney from WT mice, each antibody provided different tissue and cell localization staining. Strikingly, in all cases the staining patterns obtained for each tissue were identical for both WT and MasR-KO mice. The labeling pattern obtained after an immunohistochemical reaction depends not only on the primary antibody, but on the whole experimental procedure, that can affect final outcome including methods used for tissue fixation and antigen retrieval [41,43]. It is important to note that in the current study, experiments were performed following NLS-1531 antibody manufacturer´s instructions as AAR-013 antibody manufacturer did not provide any recommendation. In this context, the fact that the labeling pattern was identical in tissues from both WT and MasR-KO suggests that under our experimental conditions the labeling obtained with both antibodies is not specific. Still, we do not exclude the possibility that one of the antibodies used here and resulting in false-positive labelling may prove to be useful under conditions that have not been evaluated yet.

A criterion used to validate antibody specificity applied very often is the disappearance of staining in the presence of a blocking peptide, i.e the peptide against which the antibody was obtained. This control is also typically cited in antibody specification data sheets by commercial suppliers. However, current data raises questions about the validity of this test. Indeed, when AAR-013 antibody was preadsorbed with the available target peptide, the immunoreactivity of WT kidney sections was abolished. This result contrasts with the lack of specificity demonstrated in tissues from MasR-KO mice, the most stringent test of validity employed. Thus, the disappearance of staining only assures that AAR-013 antibody recognizes the peptide against which it was raised. In keeping with these results, blocking peptides had been used with several GPCR antibodies which were found not to be selective upon more stringent validation [25,27,28,30,33,34,42]. Furthermore, antigen preadsorption of AAR-013 antibody also blocked labeling of MasR-KO kidneys sections. This implies that not only on-target but also off-target binding activity of the antibody was inhibited by preadsorption with the blocking peptide. Our findings are in agreement with current reports that consider that this test is not sufficient evidence to claim specificity because it does not tell if the observed labeling represents a specific visualization of the antigen under study or it is the consequence of cross-reaction with other molecules.

To assess the possibility that obtained results were attributable to low sensitivity of the antibodies, we used HEK293T cells overexpressing c-myc-tagged human MasR. Confocal fluorescence images obtained after incubation with an anti c-myc antibody showed that a significant amount of the total MasR pool localized to the plasma membrane. The detectable non-plasma membrane-associated MasR pool probably represents newly synthesized molecules passing through the secretory pathway. Noticeable, intranuclear localization of the receptor was not observed. This cellular pattern of distribution of the MasR was similar to the findings previously reported when using MasR-GFP [44] and c-myc-MasR [20] constructs. Accordingly, we tested four MasR antibodies, all of them recommended for detection of the human receptor by IF. Noteworthy, sc-54682, sc-135063 and NLS-1531 antibodies generated a similar staining pattern to the c-myc antibody. These results indicated that these antibodies, raised against N-terminal or C-terminal domains of the MasR, were able to detect this protein at high levels in the transfected cell line. At the same time, they allowed us to verify appropriate full-length MasR protein expression in HEK 293T cells. However, sc-135063 antibody also generated widespread signals of lower intensity in cells overexpressing MasR and cells transfected with the empty vector. Thus, although the relative abundance of the target was increased by overexpressing the MasR, this antibody yielded a lower signal-to-noise ratio. Finally, AAR-013 antibody was unable to detect the specific immunoreactive MasR protein because immunofluorescence staining showed identical distribution and intensity in cells transfected with the human MasR cDNA or with the empty expression vector. We also evaluated the ability of commercial antibodies to detect MasR protein increments at different levels of overexpression by WB. The anti c-myc antibody detected a 37 kDa band, consistent with the expected size of the monomer form of the MasR. The intensity of this band increased as higher amount of DNA was transfected, providing stronger evidence that it accounts for distinct amounts of myc-tagged MasR. Additionally, a ladder of higher MW immunoreactive bands was obtained that could represent the receptor in different states of posttranslational modification (e.g. glycosylation, ubiquitination), in oligomeric forms or in aggregates formed during the denaturation step inherent to this kind of approach. A similar band pattern has been previously reported for dopamine receptors [45]. When we tested the MasR antibodies, we found that sc-54682 reproduced the band pattern obtained with the c-myc antibody. On the other hand, sc-135063 antibody revealed multiple bands close to 37 kDa with one of them of increasing intensity as higher amount of DNA was transfected but did not produce a ladder of higher MW immunoreactive bands. Besides, high unspecific staining was attained with sc-135063 antibody. Finally, AAR-013 antibody revealed a very different pattern of immunoreactivity. These results obtained by WB were in agreement with IF assays.

It is expected for MasR antibodies to detect their target over a wide range of receptor densities. We conclude that sc-54682 and sc-135063 antibodies were able to detect MasR protein of human origin when tested on a cell line with marked overexpression either by IF or by WB. However, for sc-135063 antibody, high unspecific staining was observed when tested by both techniques. NLS-1531 antibody (recommended for IF) was also able to detect human MasR by this technique. Because only sc-135063 and NLS-1531 antibodies were reported to detect MasR of mouse origin, they could be tested in mice tissues by WB and IHC, respectively. In this condition, when much lower physiological expression densities were assessed, these two antibodies were unable to detect their target. These results suggest poor sensitivity of sc-135063 and NLS-1531 antibodies for MasR. One antibody (sc-54682) could be tested only upon overexpression because of its unique reported reactivity towards human MasR. The failure of AAR-013 antibody to detect MasR either at the low expression levels occurring physiologically or at the higher expression levels observed in transfected cells does not appear to be an issue of insufficient sensitivity. As a consequence, validated antibodies able to detect endogenous MasR are still lacking.

In summary, this is the first study focused in the characterization of MasR antibodies for utilization in Western Blotting, immunofluorescence and immunohistochemistry studies. Continuous efforts are needed to develop sensitive and specific antibodies to MasR and it is recommended that the newly developed antibodies to MasR be validated before their application.

## Conclusions

We demonstrated that three commercial antibodies that were able to detect MasR protein at the high expression levels observed in a transfected cell line failed to detect this receptor in rodent tissues at physiological abundance. This finding supports the idea that specificity of an antibody cannot be generally claimed from a model system and should be evaluated not only for each application and experimental condition but also upon different expression levels of the target. Knowledge of MasR tissue distribution and abundance is a critical issue in the process of understanding its function. Our current study questions the validity of using any of these antibodies for detection of the endogenous MasR. Interpretations of previously published work based on qualitative and/or quantitative estimations of the MasR protein using these antibodies should be re-evaluated and considered with rigorous caution. Without a reliable antibody, alternative methods, such as quantitative real-time–polymerase chain reaction, Northern blot or *in situ* hybridization should be used. While specific labelled ligands have been extremely useful for studies of many endogenous GPCRs [33], binding experiments specificity using labeled Ang-(1–7) is currently under discussion [9, 18, 23, 46, 47]. Thus, antibodies are one of the best tools to study MasR localization and protein abundance. In many cases researchers must use an antibody with no reliable information regarding its usefulness for their application. It is important that the companies inform in detail the conditions in which antibodies should be used according to their application to avoid erroneous data like those exposed in these series of mentioned articles. Successful strategies to generate highly specific and broadly applicable antibodies against the MasR appear to be a high priority task to continue research on this field.

## Supporting information

S1 Fig Original uncropped and unadjusted agarose gels corresponding to Fig 2 from the main text. Ladders and their base pair lengths are shown.
(TIF)

S2 Fig Original uncropped and unadjusted blots corresponding to Fig 3 from the main text. Molecular size markers are shown. Red boxes indicate the areas included in Fig 3. WT: wild type, MasR-KO: Mas receptor knockout, X: any sample.
(TIF)

S3 Fig Original uncropped and unadjusted blots corresponding to Fig 7 from the main text. Molecular size markers are shown. Red boxes indicate the areas included in Fig 7. WT: wild type, MasR-KO: Mas receptor knockout, X: any sample.
(TIF)
