# Supplementary figures and images for: Validation of commercial Mas receptor antibodies for utilization in Western Blotting, immunofluorescence and immunohistochemistry studies
Source: PLoS One. 2017 Aug 16;12(8):e0183278. doi: 10.1371/journal.pone.0183278 (PMC5558983; doi:10.1371/journal.pone.0183278)

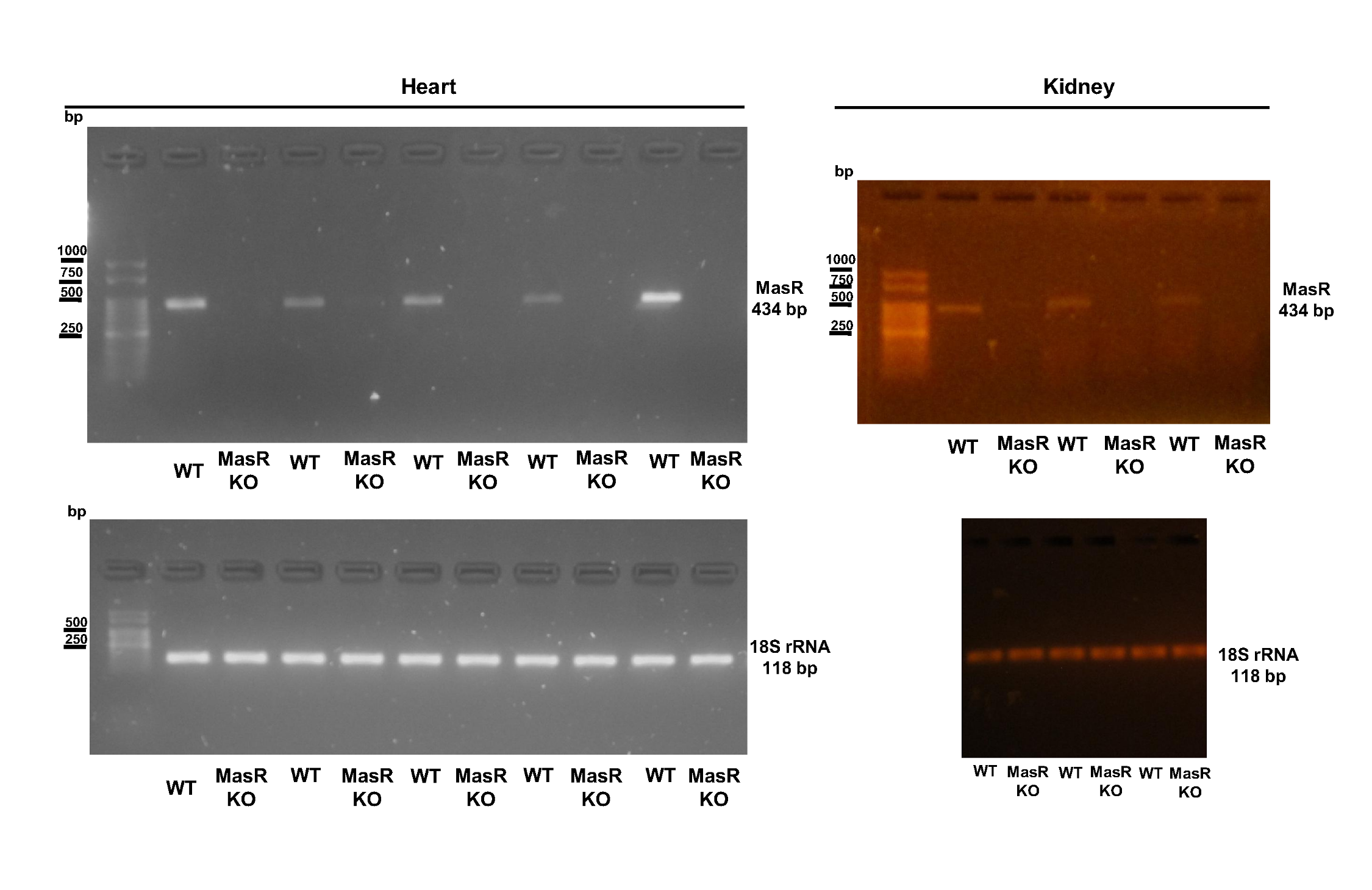

Supplement: S1 Fig — Ladders and their base pair lengths are shown. (TIF) [file pone.0183278.s001.tif]

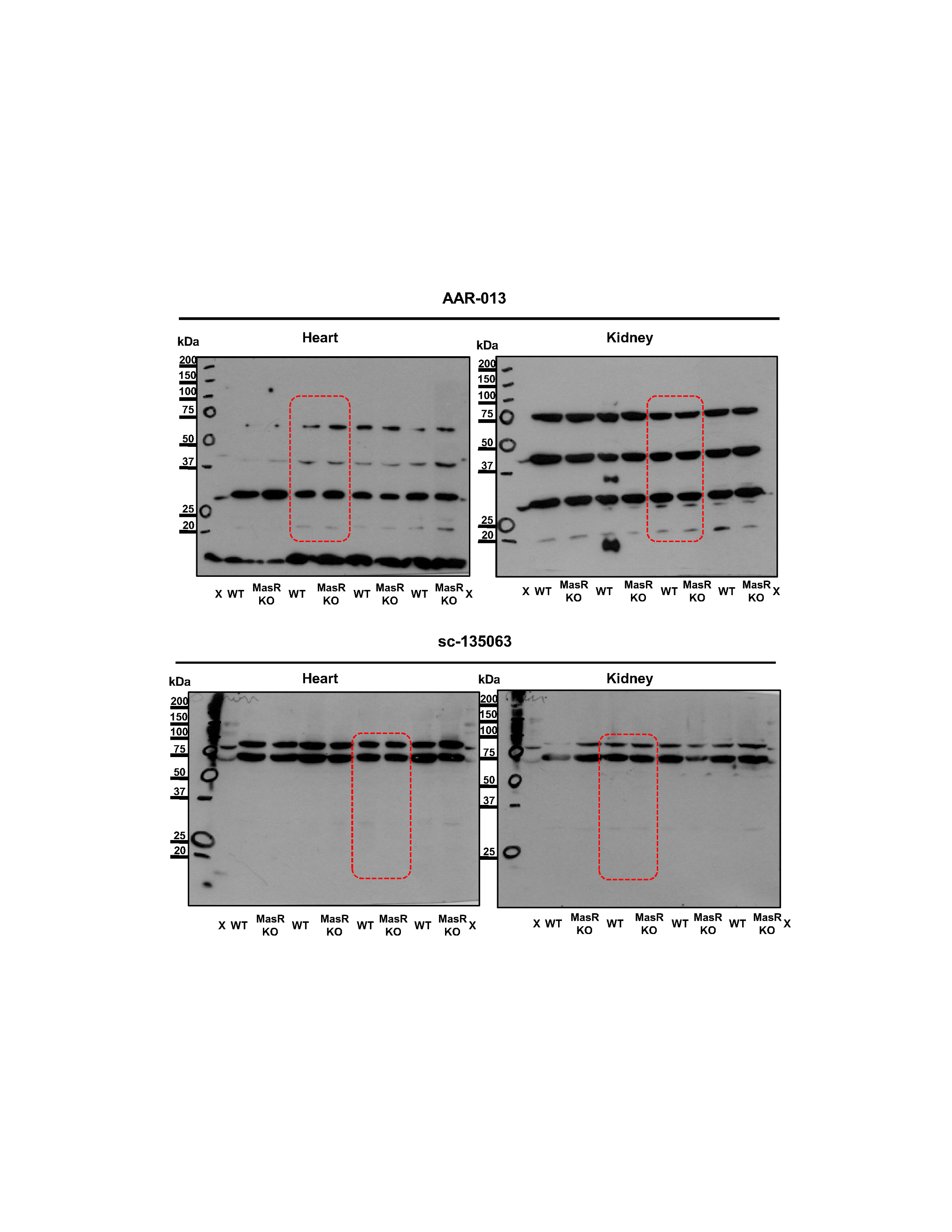

Supplement: S2 Fig — Molecular size markers are shown. Red boxes indicate the areas included in Fig 3. WT: wild type, MasR-KO: Mas receptor knockout, X: any sample. (TIF) [file pone.0183278.s002.tif]

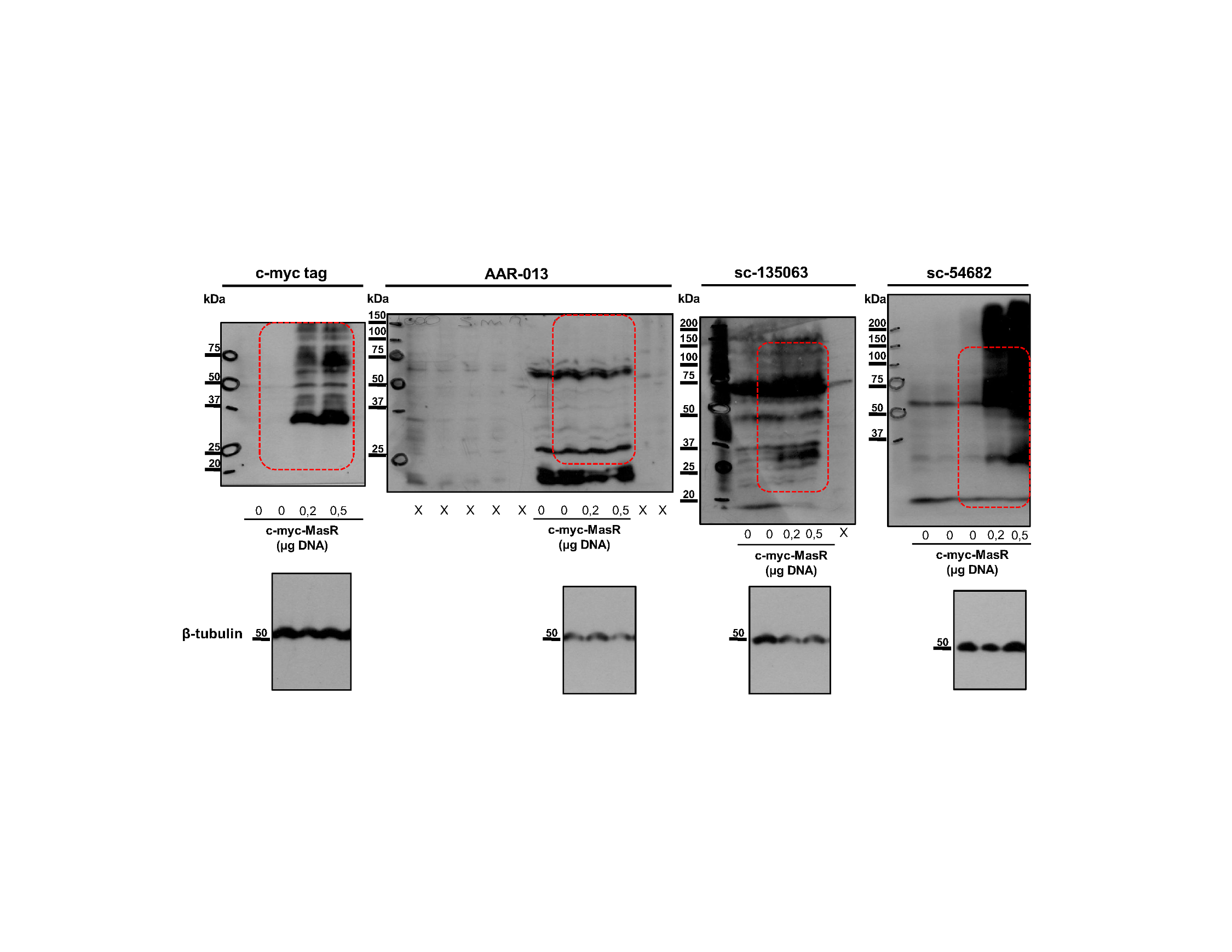

Supplement: S3 Fig — Molecular size markers are shown. Red boxes indicate the areas included in Fig 7. WT: wild type, MasR-KO: Mas receptor knockout, X: any sample. (TIF) [file pone.0183278.s003.tif]
